# When personal resources may not be enough: burnout, psychological capital, and empathy among pediatric nurses in a Chinese tertiary hospital — an explanatory sequential mixed methods study

**DOI:** 10.3389/fpubh.2026.1912710

**Published:** 2026-09-09

**Authors:** Qiong Chen, Ying-Xin Li, Xiujuan Zhang, Yanling Hu, Hongyu Huang, Yuan Li

**Affiliations:** 1 Department of Nursing, West China Second University Hospital, Sichuan University, Chengdu, China; 2 Key Laboratory of Birth Defects and Related Diseases of Women and Children (Sichuan University), Ministry of Education, Chengdu, China; 3 Department of Neonatology, West China Second University Hospital, Sichuan University, Chengdu, China; 4 College of Nursing, Silliman University, Dumaguete City, Negros Oriental, Philippines; 5 Department of Nursing, WCSUH-Tianfu/Sichuan Provincial Children’s Hospital, Meishan, China

**Keywords:** burnout, professional, empathy, mixed methods research, nursing management, pediatric nursing, psychological capital

## Abstract

**Aims:**

Healing workplaces require evidence on how personal resources are experienced under demanding clinical conditions and what forms of support nurses perceive as useful. This study examined burnout, psychological capital, and empathy among pediatric nurses in a Chinese tertiary hospital and used qualitative findings from nurses with extreme burnout profiles to clarify burnout-related support and training needs.

**Methods:**

An explanatory sequential mixed methods design was used. In the quantitative phase, 218 pediatric nurses completed an online survey including the Maslach Burnout Inventory–Human Services Survey, the Chinese Revised Nurses’ Psychological Capital Scale, and the Jefferson Scale of Empathy–Health Professionals. In the qualitative phase, 11 nurses with extreme burnout profiles participated in one 120-min focus group. Quantitative data were analyzed using descriptive statistics, unadjusted Pearson correlations, and 95% confidence intervals. Qualitative data were analyzed using inductive thematic analysis and integrated with quantitative findings through a joint display.

**Results:**

Reduced personal accomplishment was the most prevalent burnout dimension (54.6%), whereas emotional exhaustion and depersonalization were low in most participants (75.0% and 84.0%, respectively). Psychological capital was low in 76.0% of nurses and was not significantly correlated with any burnout dimension. Empathy was high in 62.0% of participants and was positively correlated with personal accomplishment (*r* = 0.405) and negatively correlated with emotional exhaustion (*r* = −0.184) and depersonalization (*r* = −0.313). Qualitative findings identified perceived support needs related to burnout recognition, psychological guidance, career development, professional enhancement, case-based learning, confidentiality, and follow-up evaluation.

**Conclusion:**

Pediatric nurses showed a burnout profile dominated by diminished personal accomplishment. The non-significant psychological capital–burnout associations suggest that personal resources alone may not fully explain burnout patterns in this single-center context. Burnout support in high-acuity pediatric settings may need to combine psychological support with career-development pathways, team learning, and reflective practice.

## 1 Introduction

Burnout is a work-related syndrome arising from chronically unmanaged occupational stress, characterized by emotional exhaustion, depersonalization, and reduced personal accomplishment (1, 2). This phenomenon represents a substantial challenge for the global nursing workforce. A meta-analysis of 113 studies involving 45,539 nurses across 49 countries reported a pooled burnout prevalence of 11.23%, with significant variation across regions and specialties (3). Burned-out nurses are more likely to leave their posts, deliver care with compromised safety climates, and contribute to higher rates of missed nursing care and adverse organizational outcomes (4, 5). At a macro level, the World Health Organization estimates that mental health conditions, including burnout-related depression and anxiety, account for an annual global productivity loss of approximately US$1 trillion, with healthcare workers among the occupational groups most affected (6).

In China, the burden of nurse burnout appears even more pronounced, with meta-analytic data indicating an overall prevalence of 64.06%, including 7.83% classified as severe (7). Recent evidence has reframed Chinese nurse burnout as a systemic problem rooted in structural, organizational, and cultural pressures rather than in individual vulnerability alone, with a large-scale mixed methods study using natural language processing of social media data describing burnout as the product of pervasive institutional conditions across the healthcare system (8). This reframing carries direct implications for nursing management research, because personal psychological resources may not remain equally protective when nurses work under persistent, institutionally embedded demands.

Pediatric nurses in tertiary Chinese hospitals occupy a particularly demanding position. They care for critically ill, developmentally vulnerable, and pre-verbal children, while simultaneously navigating parental anxiety, ethical complexities, and rapid changes in clinical acuity (9). In China, pediatric nursing is shaped by the shortage of pediatric specialists, high family expectations, and the concentration of pediatric patients in tertiary hospitals (8, 10). These contextual pressures are compounded by rotating shift work, role overload, professional-title competition, and hierarchical management structures (11). Pediatric nursing also involves intensive emotional labor, as nurses must sustain patience, compassion, and professional composure across long shifts of caring for ill children and supporting their families (12). More recent reformulations of the burnout construct further recognize that the three dimensions of burnout do not always co-occur in fixed sequence and may instead manifest as distinct profiles depending on the work context (13, 14), indicating the importance of examining burnout patterns specific to high-intensity nursing settings rather than assuming a uniform syndrome.

Psychological capital (PsyCap) and empathy are two personal resources frequently positioned as potentially protective against burnout (15, 16). PsyCap comprises hope, self-efficacy, resilience, and optimism, and is conceptualized as a state-like resource that can be developed through targeted intervention (17, 18). Within the frameworks of the Job Demands-Resources (JD-R) model and Conservation of Resources (COR) theory, PsyCap is theorized to help nurses sustain motivation, interpret stressors adaptively, and recover from cumulative resource loss (19, 20). An inverse association between PsyCap and burnout has been reported across general nursing populations, although this evidence has been generated largely in samples that may not reflect the specific demands of high-intensity tertiary pediatric units (21). Empathy, the cognitive and affective capacity to understand patients’ perspectives and respond with compassionate care, has likewise been linked to better perceived personal accomplishment and stronger therapeutic engagement, particularly in relational specialties (16, 22). Empathy holds particular significance in pediatric nursing, where care relies on understanding pre-verbal cues, building trust with anxious parents, and managing emotionally charged clinical encounters. Evidence among Chinese nurses suggests that empathy is associated with humanistic care and compassion satisfaction, but sustained relational engagement may also place nurses at risk of empathic strain when emotional recovery opportunities are limited (23, 24). Together, these resources have informed intervention approaches such as resilience training, mindset coaching, and empathy education as responses to nurse burnout (25).

However, this individual-resource assumption warrants closer examination within highly institutionalized clinical environments. Both the JD-R model and COR theory predict that when job demands remain persistently high and organizational resources are limited, the buffering capacity of personal resources may be constrained (19, 20). In Chinese tertiary pediatric nursing, where the structural and emotional demands outlined above intersect with limited career advancement and resource constraints, the roles of PsyCap and empathy may differ from what evidence in less constrained settings suggests (8).

Existing research has examined burnout, PsyCap, and empathy mainly as parallel correlates, without integrating qualitative accounts that could clarify how nurses themselves understand burnout-related support needs within specific work systems (21). Qualitative studies have described nurses’ burnout experiences in depth but rarely link these accounts back to a measurable workforce-level burnout profile (26). An explanatory sequential mixed methods design is therefore well suited to this question, as the qualitative phase can explain and contextualize quantitative patterns and inform management-relevant training implications (27, 28). Accordingly, this study examined burnout, PsyCap, and empathy among pediatric nurses in a tertiary hospital in Sichuan Province, China. The study addressed three questions: What are the levels of burnout, PsyCap, and empathy among pediatric nurses; how are PsyCap and empathy associated with the three dimensions of burnout; and what burnout-related support and training needs are identified by nurses with extreme burnout profiles?

## 2 Methods

### 2.1 Study design

This study used an explanatory sequential mixed methods design comprising a quantitative phase followed by a qualitative phase, with integration of findings through a joint display (27, 28). The quantitative phase characterized the levels and unadjusted inter-correlations of burnout, PsyCap, and empathy and identified nurses with extreme burnout profiles. The qualitative phase then drew on focus group data from extreme-case participants to explore burnout-related support and training needs and to contextualize the quantitative findings. Reporting followed the GRAMMS guidelines for mixed methods research (29).

### 2.2 Setting and participants

The study was conducted in a tertiary Grade A women’s and children’s hospital in Sichuan Province, southwest China. The hospital is a regional referral center that delivers comprehensive pediatric and obstetric services, with high patient volumes and continuous 24-h care across general pediatric wards, the neonatal intensive care unit (NICU), and the pediatric intensive care unit (PICU). Pediatric nursing care in this setting involves rotating shifts, frequent family communication, and high-acuity transitions, which together provide an information-rich context for examining burnout in tertiary pediatric nursing practice.

Eligible participants for the quantitative phase were registered nurses who (1) were employed in pediatric departments of the participating hospital, (2) had at least 1 year of clinical nursing experience, and (3) provided informed consent for voluntary participation. Nurses on short-term external rotations or those on extended leave during the data collection period were excluded. Of the 466 pediatric nurses employed at the hospital, 408 met the inclusion criteria. The required sample size was calculated using Slovin’s Formula at a 5% margin of error, yielding a minimum of 201 participants (30). Accounting for potential non-response and invalid questionnaires, recruitment was extended to 220 nurses, and a final sample of 218 valid responses was obtained. Eligible nurses were selected through simple random sampling using a random number generator, with department-level allocation proportional to each unit’s nursing workforce size.

For the qualitative phase, purposive extreme-case sampling was used (31). After the quantitative survey was scored, nurses were screened using prespecified cut-off values for low personal accomplishment (MBI-HSS score ≤33), high emotional exhaustion (≥27), and high depersonalization (≥10), with all three criteria required for eligibility (32). Eligible nurses were invited individually by the research team and provided written informed consent prior to the focus group.

### 2.3 Quantitative instruments

The online questionnaire comprised a demographic section and three validated self-report scales. Demographic items captured sex, age, educational background, current department, years of nursing experience, professional title, position, monthly income, and marital status.

Burnout was assessed using the Maslach Burnout Inventory–Human Services Survey (MBI-HSS), originally developed by Maslach and Jackson (32) and widely used in nursing and Chinese occupational research (33). The 22-item instrument measures three subscales: emotional exhaustion (9 items), depersonalization (5 items), and personal accomplishment (8 items), each rated on a 7-point frequency scale from 0 (*never*) to 6 (*every day*). Following the MBI manual, subscale scores were analyzed separately and classified into low, moderate, and high burnout levels: emotional exhaustion (0–18/19–26/27–54), depersonalization (0–5/6–9/10–30), and personal accomplishment (40–48/34–39/0–33), for which lower scores indicate reduced personal accomplishment and greater burnout (32). The Chinese MBI-HSS has shown acceptable psychometric properties in relevant occupational samples (34). In this sample, Cronbach’s α was 0.795 for all items, and subscale α values were 0.914 for emotional exhaustion, 0.818 for depersonalization, and 0.765 for personal accomplishment.

PsyCap was measured using the Chinese version of Nurses’ Psychological Capital Scale (RNPCS), adapted from the original Psychological Capital Questionnaire developed by Luthans and colleagues (17) and revised for the Chinese nursing context by Luo and Hao (35). The 20-item instrument covers four subscales: self-efficacy (6 items), hope (6 items), resilience (5 items), and optimism (3 items), each rated on a 6-point Likert scale from 1 (*strongly disagree*) to 6 (*strongly agree*). Total scores range from 20 to 120, with higher scores indicating greater PsyCap. For descriptive purposes, scores were classified as low (20–70), moderate (71–95), or high (96–120), corresponding to item-mean categories applied in previous Chinese nursing studies (36). The RNPCS has shown acceptable psychometric properties in Chinese nursing samples (35). In this sample, Cronbach’s α for the total scale was 0.964.

Empathy was measured using the Chinese version of the Jefferson Scale of Empathy–Health Professionals (JSE-HP), derived from the Jefferson Scale of Physician Empathy originally developed by Hojat et al. (37, 38) and subsequently translated and validated for Chinese healthcare populations (39). The 20-item instrument covers three dimensions: perspective taking (5 items), compassionate care (10 items), and standing in the patient’s shoes (5 items), each rated on a 7-point Likert scale, with 10 negatively worded items reverse-scored. Total scores range from 20 to 140, with higher scores indicating greater empathy, and were classified as low (20–80), moderate (81–110), or high (111–140) (37, 38). The Chinese JSE-HP has shown acceptable psychometric properties in healthcare professional samples (39). In this sample, Cronbach’s α for the total scale was 0.909.

### 2.4 Quantitative data collection and analysis

After ethical approval and administrative permission, the questionnaire was distributed electronically using the Wenjuanxing platform.^1^ A study information sheet preceded the questionnaire and described the study purpose, voluntary participation, anonymity, data confidentiality, and the right to withdraw without consequence. Completion of the questionnaire was treated as informed consent for the quantitative phase. To safeguard data quality, IP addresses were restricted to one response per device, no identifying information was collected, and participation was independent of supervisory relationships.

Data were analyzed using IBM SPSS Statistics version 26.0. Descriptive statistics summarized participant characteristics and scale scores, with continuous variables reported as means and standard deviations and categorical variables as frequencies and percentages. Unadjusted Pearson product–moment correlation coefficients were used to examine the associations of PsyCap and empathy with the three burnout dimensions, as well as the association between PsyCap and empathy. Ninety-five percent confidence intervals (CIs) for correlation coefficients were calculated using Fisher’s z transformation. A two-tailed *p* value of less than 0.05 was considered statistically significant. Missing data were minimal due to forced-response questionnaire settings, and complete-case analysis was used.

### 2.5 Qualitative data collection and analysis

A single in-person focus group was conducted in a quiet, private meeting room within the hospital, lasting approximately 120 min. The discussion was facilitated by the principal investigator (a doctorally prepared female research nurse with prior qualitative training and no line-management relationship to participants); a second researcher acted as note-taker and observer. To support psychological safety and reduce power-related pressure, the facilitator reiterated confidentiality, voluntary participation, the right not to answer questions, and de-identified reporting; participants were reminded that their comments would not affect employment or evaluation. The semi-structured interview guide (Supplementary File 1) focused on nurses’ preferences for burnout-related support and training. Discussions were audio-recorded with participants’ consent and transcribed verbatim.

Transcripts were analyzed using inductive thematic analysis based on Braun and Clarke’s six-phase framework: data familiarization, generation of initial codes, searching for themes, reviewing themes, defining and naming themes, and producing the report (40). Two researchers independently coded the transcripts using NVivo version 12.0, then discussed initial codes and candidate themes to refine the coding framework; a third researcher was consulted to review unresolved interpretive differences. Themes were generated inductively from the focus group data, and were subsequently examined alongside the quantitative findings on burnout, PsyCap, and empathy during the integration phase. Methodological rigor was supported through prolonged engagement with the data, peer debriefing, an audit trail of analytical decisions, member checking with three participants, and a reflexivity journal in which researchers documented their prior views on burnout-related support, training needs, and pediatric nursing practice (41). Following recommendations for cross-language qualitative research (42), all participant quotations were edited for English-language clarity by a bilingual research team member, with edits limited to grammatical and idiomatic correction; original meanings, emphases, and reasoning were preserved and verified against the audio transcripts.

### 2.6 Mixed methods integration

Integration occurred at two principal points following the framework of Fetters and colleagues (43). At the sampling level, qualitative participants were drawn from the quantitative dataset based on extreme burnout profiles. This connecting strategy allowed the qualitative phase to explore support and training needs among nurses with the most pronounced burnout profiles, thereby adding contextual depth to the quantitative findings. At the interpretation level, quantitative and qualitative findings were merged in a joint display to generate integrated inferences. The joint display was structured around the constructs measured in the quantitative phase, with each construct mapped to relevant qualitative themes to identify convergence/confirmation, expansion, or divergence/discordance between strands.

### 2.7 Ethics statement

Ethical approval was obtained from the University Research Ethics Committee of Silliman University (approval date: January 26, 2024). Because the study formed part of the first author’s doctoral research at Silliman University, ethical review was obtained from this institution, and data collection in China was conducted after administrative permission from the participating hospital. The study complied with the Declaration of Helsinki and relevant institutional requirements. All participants provided informed consent; survey completion indicated electronic consent, and written consent was obtained before the focus group. Participation was voluntary, withdrawal had no employment consequences, and data were reported anonymously and stored securely.

## 3 Results

### 3.1 Sample characteristics

A total of 218 pediatric nurses completed the survey. The sample was predominantly female (92.7%), held a bachelor’s degree (83.5%), and was concentrated in high-acuity settings, with 47.7% working in the NICU and 9.6% in the PICU. Detailed characteristics are presented in Table 1.

**Table 1:** Demographic characteristics of participants (*N* = 218).

Characteristic	Category	*n*	%
Sex	Male	16	7.3
	Female	202	92.7
Educational level	Three-year college diploma	15	6.9
	Bachelor’s degree	182	83.5
	Master’s or doctoral degree	21	9.6
Department	Child healthcare	9	4.1
	Pediatric neurology	11	5.0
	Pediatric respiratory	11	5.0
	Pediatric gastroenterology	10	4.6
	Pediatric nephrology	9	4.1
	Pediatric hematology	20	9.2
	Pediatric infection	16	7.3
	Pediatric surgery	7	3.2
	NICU	104	47.7
	PICU	21	9.6
Years of nursing experience	1–4	104	47.7
	5–10	58	26.6
	≥10 years	56	25.7
Professional title	Nurse	141	64.7
	Supervising nurse	72	33.0
	Associate chief nurse or above	5	2.3
Employment type	Contract-based	193	88.5
	Outsourced	24	11.0
	Permanent appointment	1	0.5
Monthly income (RMB)	<6,000	23	10.6
	6,000–9,999	69	31.7
	10,000–19,999	113	51.8
	20,000 or more	13	6.0
Marital status	Never married	90	41.3
	Married	123	56.4
	Divorced or widowed	5	2.3

NICU, neonatal intensive care unit; PICU, pediatric intensive care unit; RMB, renminbi (Chinese yuan). Percentages may not sum to 100.0 due to rounding.

### 3.2 Levels of burnout, psychological capital, and empathy

Reduced personal accomplishment was the most prominent burnout dimension in this sample. More than half of the nurses (54.6%, *n* = 119) fell in the low personal accomplishment range. In contrast, the majority reported low levels of emotional exhaustion (74.8%, *n* = 163) and low depersonalization (83.9%, *n* = 183). Mean subscale scores were 30.91 (SD = 9.06) for personal accomplishment, 14.32 (SD = 3.80) for emotional exhaustion, and 2.74 (SD = 4.28) for depersonalization. PsyCap was low in 75.7% (*n* = 165) of nurses, moderate in 18.0% (*n* = 39), and high in only 6.4% (*n* = 14), with a mean total score of 63.19 (SD = 18.05). Empathy showed an inverse distribution: 62.4% (*n* = 136) of nurses scored in the high range, 31.7% (*n* = 69) in the moderate range, and 6.0% (*n* = 13) in the low range, with a mean total score of 113.52 (SD = 16.22). Scale-level findings are summarized in Table 2.

**Table 2:** Levels of burnout, psychological capital, and empathy (*N* = 218).

Variable	Score range	Level	*n*	%
Burnout (MBI-HSS)				
Personal accomplishment	40–48	Low burnout	41	18.8
	34–39	Moderate burnout	58	26.6
	0–33	High burnout	119	54.6
Mean = 30.91, SD = 9.06				
Emotional exhaustion	0–18	Low burnout	163	74.8
	19–26	Moderate burnout	27	12.4
	27–54	High burnout	28	12.8
Mean = 14.32, SD = 3.80				
Depersonalization	0–5	Low burnout	183	83.9
	6–9	Moderate burnout	15	6.9
	10–30	High burnout	20	9.2
Mean = 2.74, SD = 4.28				
Psychological capital (RNPCS)				
Total score	20–70	Low	165	75.7
	71–95	Moderate	39	17.9
	96–120	High	14	6.4
Mean = 63.19, SD = 18.05				
Empathy (JSE-HP)				
Total score	20–80	Low	13	6.0
	81–110	Moderate	69	31.7
	111–140	High	136	62.4
Mean = 113.52, SD = 16.22				

MBI-HSS, Maslach Burnout Inventory–Human Services Survey; RNPCS, Chinese Revised Nurses’ Psychological Capital Scale; JSE-HP, Jefferson Scale of Empathy–Health Professionals; SD, standard deviation. For personal accomplishment, lower scores indicate greater burnout.

### 3.3 Correlations among burnout, psychological capital, and empathy

Pearson correlation analyses revealed a notable divergence between the two personal resources examined. PsyCap was not significantly associated with any burnout dimension. The correlation coefficients were very small for personal accomplishment (*r* = −0.005, 95% CI: −0.138 to 0.128, *p* = 0.941), emotional exhaustion (*r* = −0.052, 95% CI: −0.184 to 0.081, *p* = 0.445), and depersonalization (*r* = −0.038, 95% CI: −0.170 to 0.095, *p* = 0.577). PsyCap and empathy showed a small, non-significant positive association (*r* = 0.126, 95% CI: −0.007 to 0.255, *p* = 0.064). In contrast, empathy showed a more favorable pattern of unadjusted associations with burnout dimensions. Higher empathy was moderately correlated with higher personal accomplishment (*r* = 0.405, 95% CI: 0.288 to 0.510, *p* < 0.001) and weakly to moderately correlated with lower emotional exhaustion (*r* = −0.184, 95% CI: −0.309 to −0.052, *p* = 0.006) and lower depersonalization (*r* = −0.313, 95% CI: −0.428 to −0.188, *p* < 0.001). Detailed results are presented in Table 3.

**Table 3:** Pearson correlations among burnout dimensions, psychological capital, and empathy (*N* = 218).

Variable pair	*r*	95% CI	*p* value
Burnout dimensions and PsyCap			
Personal accomplishment × PsyCap	−0.005	−0.138 to 0.128	0.941
Emotional exhaustion × PsyCap	−0.052	−0.184 to 0.081	0.445
Depersonalization × PsyCap	−0.038	−0.170 to 0.095	0.577
Burnout dimensions and Empathy			
Personal accomplishment × Empathy	0.405	0.288 to 0.510	<0.001
Emotional exhaustion × Empathy	−0.184	−0.309 to −0.052	0.006
Depersonalization × Empathy	−0.313	−0.428 to −0.188	<0.001
PsyCap and empathy			
PsyCap × Empathy	0.126	−0.007 to 0.255	0.064

CI, confidence interval. Two-tailed *p* values are reported. Confidence intervals were calculated using Fisher’s z transformation.

### 3.4 Qualitative findings from extreme-burnout participants

**Figure 1:**
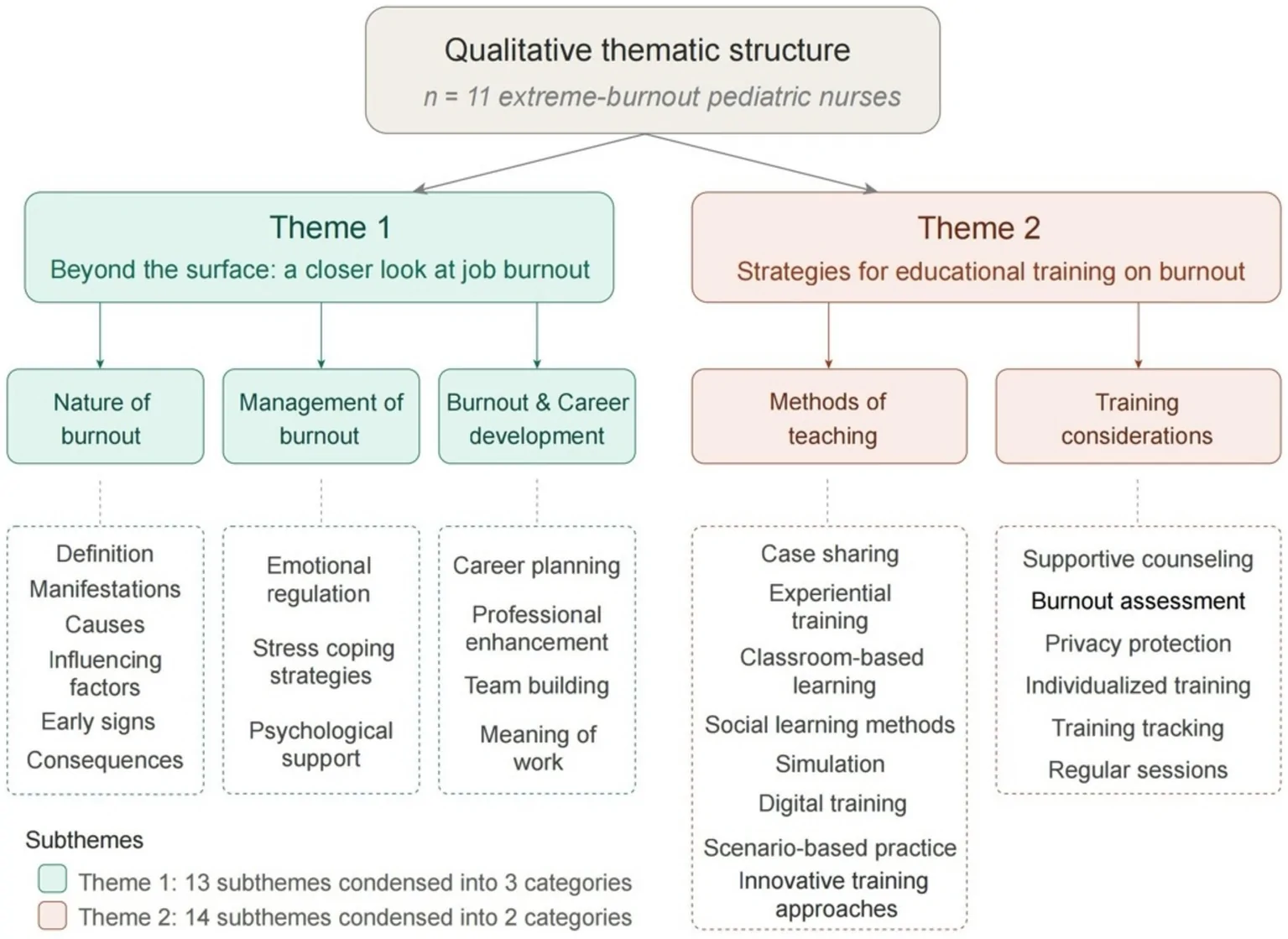
Qualitative thematic structure derived from focus group data.

Among the 218 respondents, 11 met all three prespecified extreme-burnout criteria, and all 11 participated in one 120-min focus group. Their individual demographic characteristics are presented in Supplementary File 2. Compared with the full sample, the subgroup was descriptively similar in education level but included more nurses with fewer than 10 years of experience and more married nurses. Inductive thematic analysis yielded 27 subthemes, condensed into five thematic categories, which were further synthesized into two central themes: *Beyond the surface: a closer look at job burnout* and *Strategies for educational training on burnout*. The full thematic structure is presented in Figure 1.

#### 3.4.1 Theme 1: beyond the surface — a closer look at job burnout

Participants described burnout-related support needs that went beyond general stress relief and included understanding burnout, managing psychological distress, strengthening professional identity, and planning career development. Three thematic categories were generated under this theme: the nature of burnout, the management of burnout, and burnout in relation to career development.

In describing the nature of burnout, participants emphasized the importance of understanding its definition, manifestations, causes, and consequences as foundations for self-recognition. They expected training to help nurses recognize early warning signs and distinguish burnout from related psychological distress:

“*The training I’d like to receive may include: What are the signs of burnout? What are the causes of burnout? What are the peak times for burnout? Is burnout a mental illness or a predisposition to depression?*” (P3)

“*I think we should learn the definition and common manifestations of burnout, such as physiological reactions, psychological manifestations, and behavioral changes, as well as its influencing factors and the harms and consequences it can bring.*” (P6)

When discussing the management of burnout, participants emphasized emotional regulation, stress release strategies, and psychological guidance. They also expected training to address coping at both individual and leadership levels:

“*How to make the body relax when tired from work, how to control emotions and release pressure during work.*” (P2)

“*The training should also cover coping strategies for burnout, both at the individual level and at the leadership level.*” (P6)

“*I think the training program should at least include psychological guidance and counseling support*.” (P8)

A particularly important finding was that participants linked burnout-related training to career development and professional value, rather than viewing it only as psychological stress management. Participants suggested that training should include professional development, career planning, professional value, communication skills, and team building:

“*The content should include improving staff interpersonal relationships, learning new knowledge, new ideas, and new technologies, as well as preparation for professional title promotion.*” (P1)

“*The program should cover what methods can increase nurses’ sense of professional honor and pride, how to improve our professional quality, and how to help nurses build a clear career development plan.*” (P2)

“*What I want from training is not only stress management. It should include career development, career value, sense of achievement, communication skills, and even interest cultivation.*” (P8)

“*For me, the most important thing in training is finding the meaning of work and reconnecting with the value of what we do*.” (P11)

Across the three categories, participants described burnout training needs that extended beyond individual coping skills, including burnout recognition, psychological guidance, career development, professional enhancement, team building, and support from both individual and leadership levels.

#### 3.4.2 Theme 2: strategies for educational training on burnout

Participants discussed how burnout-related training should be designed and delivered. Beyond training content, they emphasized that training needed to be feasible for shift-working nurses, clinically relevant, interactive, and supported by follow-up. Two thematic categories were generated under this theme: methods of teaching and training considerations.

Preferred methods of teaching clustered around active and case-based formats rather than traditional didactic lectures. Suggested formats included case sharing, experiential training, group discussion, simulation, online training, and outdoor team-building activities:

“*There can be online training and case discussion, that is, through a few typical cases of burnout to change the attitude of burnout*.” (P5)

“*My personal favorite is open-ended and flexible training. For professional training, outdoor group activities are very meaningful and well worth recommending.*” (P7)

“*My suggestion is to use scenario simulation, not traditional teaching methods*; *the training methods I recommend are tea parties, book clubs, meditation sessions, and outdoor exercise*.” (P10)

Regarding training considerations, participants emphasized confidentiality, individualized support, regular sessions, follow-up evaluation, and opportunities for self-directed learning. Privacy protection was particularly important:

“*Regular psychological online interviews should be arranged, but they must be anonymous. When personal privacy is involved, a comfortable, closed space should be provided for communication. Burnout training should focus on soothing nurses’ emotions, not on adding empty motivational talk*.” (P3)

“*Hospitals need regular job training and regular symposiums; maybe once every six months would be ideal.*” (P2)

“*Burnout is not just about completing the training program. We should pay more attention to following up on its effects, and adjust the program in time according to those effects.*” (P4)

“*I think the reasons why burnout occurs may be the same or different for each person. So the training strategy should combine focused lectures for the shared issues with workshops or interviews for the individual ones*.” (P8)

“*It would also help if the department could provide some self-help or motivational books that nurses can read during their breaks*.” (P10)

Participants emphasized that burnout-related training should be practical, confidential, continuous, and grounded in real pediatric nursing scenarios. They preferred a blended approach that combined shared education, individualized psychological support, follow-up assessment, and clinically relevant learning formats.

### 3.5 Mixed methods integration of quantitative and qualitative findings

Integration of the quantitative and qualitative strands generated four management-relevant interpretive inferences. First, the high prevalence of reduced personal accomplishment in the quantitative phase aligned with participants’ qualitative emphasis on career development, professional value, professional enhancement, and the search for meaning at work, supporting the relevance of personal accomplishment as a key management concern in this setting. Second, the low PsyCap levels and absence of any significant linear association with burnout aligned with participants’ requests for organizational and career-level support rather than individual coping training, suggesting, as an inference from qualitative accounts and contextual evidence, that individual psychological resources may need to be considered alongside broader work conditions. Third, the high empathy levels and empathy-burnout associations aligned with participants’ preference for case-based, experiential, and discussion-based learning, suggesting hypotheses for future training research focused on preserving relational strengths while providing structured opportunities for reflection and emotional support. Fourth, the deliberate selection of extreme-burnout cases for the focus group highlighted a training gap, with participants describing the need for structured, ongoing programs rather than *ad hoc* lectures. The full joint display is presented in Table 4.

**Table 4:** Joint display integrating quantitative and qualitative findings.

Construct/domain	Quantitative finding (*n* = 218)	Qualitative finding (*n* = 11)	Fit between strands	Integrated inference for nursing management
Personal accomplishment	Reduced personal accomplishment was the most prevalent burnout dimension. A total of 54.6% of nurses (*n* = 119) scored in the high-burnout range for this dimension, with a mean score of 30.91 (SD = 9.06).	Participants emphasized training needs related to career development, professional value, professional enhancement, and the search for meaning at work, including the need to reconnect with “the meaning of work” (P11).	Convergence + expansion	Diminished personal accomplishment could be treated as a key management concern in this setting. Routine burnout monitoring could incorporate perceived recognition, professional confidence, and career-development needs alongside conventional exhaustion indicators.
Emotional exhaustion	The majority of nurses reported low emotional exhaustion (74.8%, *n* = 163), with a mean score of 14.32 (SD = 3.80). Only 12.8% reached the high-burnout range.	Participants requested training in emotional regulation, stress release, and psychological guidance, including “how to control emotions and release pressure” (P2).	Expansion	Although emotional exhaustion was not the dominant burnout dimension, nurses still identified emotional regulation and psychological guidance as important support needs. Future training could include practical strategies for emotional recovery and stress management.
Depersonalization	Most nurses reported low depersonalization (83.9%, *n* = 183), with a mean score of 2.74 (SD = 4.28). Only 9.2% reached the high-burnout range.	Participants did not emphasize detached or cynical attitudes toward patients. Instead, their training suggestions included communication skills, professional value, and support for maintaining meaningful pediatric care.	Convergence + expansion	Relational engagement appeared largely preserved in this sample. Future management strategies could protect this relational capacity through communication training, reflective practice, and compassion fatigue prevention.
Psychological capital	Psychological capital was low in 75.7% of nurses (*n* = 165; mean = 63.19, SD = 18.05) and showed no significant association with any burnout dimension (*r* values from −0.005 to −0.052; all *p* > 0.40).	Participants’ support needs extended beyond individual coping to include psychological guidance, leadership-level coping strategies, career development, professional enhancement, structured learning, and follow-up assessment.	Expansion	The non-significant PsyCap–burnout associations, together with nurses’ broader support needs, suggest that PsyCap training may be better evaluated not as a stand-alone intervention but as one component of a wider support program that includes psychological support, professional development, team learning, and managerial support.
Empathy	Empathy was high in 62.4% of nurses (*n* = 136; mean = 113.52, SD = 16.22) and was positively correlated with personal accomplishment (*r* = 0.405, *p* < 0.001) and negatively correlated with emotional exhaustion (*r* = −0.184, *p* = 0.006) and depersonalization (*r* = −0.313, *p* < 0.001).	Participants valued case-based, experiential, discussion-based, and simulation-based learning formats, which can support reflection on real pediatric communication and care situations.	Convergence + expansion	Empathy may be regarded as a preserved professional resource in this setting. Future training could protect nurses’ relational strengths through reflective learning, peer discussion, emotional boundary support, and compassion fatigue prevention.
Training content preferences	Not directly assessed quantitatively. The qualitative sample was selected from nurses with extreme burnout profiles identified in the survey.	Participants requested training content covering burnout recognition, psychological guidance, career development, professional enhancement, team building, communication skills, and professional value. Career-related content was a prominent part of their support needs.	Expansion	Future training content could include three core elements: burnout literacy and early-warning recognition, psychological guidance and emotional regulation, and career-development support with professional identity building.
Training delivery preferences	Not directly assessed quantitatively.	Participants preferred active and clinically relevant formats, including case sharing, experiential training, group discussion, simulation, online or digital training, and outdoor team-building. They also emphasized confidentiality, individualized follow-up, regular sessions, and burnout assessment.	Expansion	A feasible future training program could combine case-based workshops, simulation, peer discussion, online learning, confidential support, and follow-up assessment, grounded in real pediatric nursing scenarios.
Overall integration	Quantitative profile: reduced personal accomplishment was the most prevalent burnout dimension; PsyCap was not significantly associated with burnout; empathy was associated with a more favorable burnout profile.	Qualitative profile: nurses with extreme burnout profiles identified support needs that extended beyond individual coping and included psychological guidance, career development, professional enhancement, team building, confidentiality, and practical training formats.	Integration	Burnout management in this tertiary pediatric setting may need to move beyond stand-alone resilience education and combine individual psychological support with professional development, team learning, managerial support, and reflective practice.

CI, confidence interval; NICU, neonatal intensive care unit; PICU, pediatric intensive care unit; PsyCap, psychological capital; SD, standard deviation. Fit describes the relationship between quantitative and qualitative findings following Fetters et al. (43): convergence/confirmation indicates that the two strands point in the same direction; expansion indicates that one strand extends or contextualizes the other; divergence/discordance indicates that the two strands point in different directions. No clear divergence/discordance was identified in the present integration.

## 4 Discussion

This study examined burnout, PsyCap, and empathy among pediatric nurses in a tertiary Chinese hospital and yielded three findings that warrant careful interpretation. First, reduced personal accomplishment was substantially more prevalent than emotional exhaustion or depersonalization, identifying a distinctive burnout profile in this high-acuity pediatric setting. Second, PsyCap, despite being theorized as a state-like protective resource, showed no measurable association with any burnout dimension. Third, empathy was associated with better perceived accomplishment and with lower emotional exhaustion and depersonalization. The qualitative phase clarified how nurses with extreme burnout profiles understood burnout-related support needs, including burnout recognition, psychological guidance, career development, professional enhancement, team building, and practical training formats. Taken together, these findings suggest that in this tertiary pediatric setting, burnout was most visible as diminished professional accomplishment, while the role of personal resources appeared to depend on the broader conditions under which nurses worked and received support.

### 4.1 Diminished personal accomplishment as the most prominent burnout dimension

The prominence of reduced personal accomplishment in this sample (54.6%), against a backdrop of comparatively low emotional exhaustion and low depersonalization, departs from the conventional emphasis on emotional exhaustion as the core dimension of burnout (44). This pattern echoes critical care evidence: an umbrella review of 14 systematic reviews and meta-analyses found that ICU nurses had the highest pooled prevalence of low personal accomplishment across work units (45). This comparison is relevant because nearly 60% of participants in the present study worked in NICU or PICU. Pediatric critical care requires continuous surveillance, technically demanding care, rapid responses to clinical deterioration, and communication with highly anxious families. Yet patient outcomes may remain uncertain despite intensive nursing effort, particularly when children are critically ill, developmentally vulnerable, or medically complex. For pediatric nurses, this creates a high-effort but often low-visibility work pattern, in which substantial clinical and emotional labor may not be matched by immediate recovery, clear feedback, or sufficient professional recognition. Such a mismatch between sustained effort and visible recovery may weaken nurses’ sense of professional efficacy and accomplishment. Although emotional exhaustion has often been treated as the central dimension of burnout (46), profile-based approaches recognize that emotional exhaustion, depersonalization, and reduced personal accomplishment do not always co-occur with the same intensity (13). The present findings therefore add to this literature by showing that reduced accomplishment may be the most salient dimension in a pediatric nursing group that otherwise reports relatively low emotional exhaustion and depersonalization.

The qualitative data further contextualize this pattern. Participants did not simply describe themselves as fatigued; they requested training that would help them understand burnout, manage psychological distress, strengthen professional identity, and plan career development. Several participants explicitly mentioned career planning, professional honor, professional quality, communication skills, and the need to reconnect with the meaning of work. These accounts are consistent with the quantitative prominence of reduced personal accomplishment, as both point to nurses’ concern with professional value and development rather than only with emotional depletion. Similar concerns about diminished professional value and stagnation have been described in recent analyses of Chinese nurse discourse (8). For nursing managers, this finding suggests that burnout monitoring in pediatric units could avoid focusing exclusively on exhaustion or turnover intention. Routine staff conversations and well-being assessments could also include perceived achievement, professional confidence, career-development needs, and opportunities for clinical growth.

### 4.2 Psychological capital and the limits of individual-resource explanations

The absence of significant linear associations between PsyCap and any burnout dimension in this sample contrasts with broader nursing evidence showing an inverse relationship between PsyCap and burnout (21). This unadjusted finding does not refute the broader evidence base; rather, it indicates that the expected buffering role of PsyCap was not observed in this specific tertiary pediatric sample. This pattern may be interpreted in light of the boundary conditions specified by both the JD-R model and COR theory. Both frameworks suggest that when job demands remain persistently high and organizational resources are limited, personal resources may be less likely to translate into lower burnout, because individual reserves are repeatedly consumed by the strain process rather than translated into sustained engagement (19, 20). Although organizational demands and resources were not directly measured, the predominance of NICU and PICU work (57.3%) and the high prevalence of contractual rather than permanent employment (88.5%) point to a demanding and potentially unstable work context. In such a context, hope, self-efficacy, resilience, and optimism may remain valuable but may be insufficient on their own without adequate organizational and professional support.

The qualitative findings helped contextualize this interpretation by showing that nurses’ support needs extended beyond individual coping to include psychological guidance, leadership-level coping strategies, career development, professional enhancement, structured learning, and follow-up assessment. Rather than emphasizing resilience training or mindset coaching alone, participants highlighted burnout recognition, psychological support, career planning, professional value, team building, case-based learning, simulation, and continued evaluation. These accounts indicate that nurses with extreme burnout profiles desired support as a broader professional and organizational process rather than individual coping instruction alone. Taken together, the non-significant PsyCap-burnout correlations and the qualitative emphasis on broader support needs suggest that future support strategies could avoid relying solely on individual psychological resources. PsyCap training may still hold value, but future intervention research should test whether it is more acceptable or effective when embedded within a multi-level program that also addresses psychological support, professional development, team learning, and managerial support. This interpretation aligns with recent calls in nursing research for more context-sensitive models of how personal resources operate within specific organizational conditions, rather than treating them as uniformly operating individual traits (47).

### 4.3 Empathy as a preserved but vulnerable professional resource

In contrast to PsyCap, empathy was associated with better perceived accomplishment and with lower emotional exhaustion and depersonalization. One reason for this divergence may lie in the situational specificity of the two resources. PsyCap is largely an internal, self-referential construct that depends on supportive feedback loops from the work environment to be reinforced over time (48), whereas empathy is enacted in concrete relational encounters with patients and families and may be sustained through the routine clinical interactions of pediatric nursing practice (22). In this setting, the relational demands of pediatric care may have provided a continuing context in which empathy could function and support personal accomplishment.

At the same time, the coexistence of high empathy and reduced personal accomplishment is noteworthy. It suggests that nurses may continue to care compassionately for children and families while feeling that their own professional growth, recognition, or achievement is limited. This pattern may reflect a mismatch between high relational investment and insufficient professional recognition, rather than a lack of compassion or commitment. In other words, relational engagement appears to be preserved even when professional efficacy is weakened. This interpretation is consistent with recent qualitative evidence from mainland China that locates nurse burnout within broader structural and organizational conditions rather than individual maladaptation alone (8). This observation parallels the qualitative finding that participants valued case-based, experiential, and reflective learning formats over didactic content. Sustained exposure to children’s suffering and family distress carries a recognized risk of empathic strain and compassion fatigue when emotional boundaries, peer debriefing, and recovery opportunities are inadequate (16). Recent intervention studies in PICUs suggest that structured stress debriefings and reflective practice forums may reduce compassion fatigue and support compassion satisfaction in nurses working with critically ill children (49, 50). The qualitative emphasis on confidentiality is also relevant here. For nurses experiencing burnout, private and psychologically safe training spaces may lower the perceived risk of disclosure and make reflective discussion more acceptable. Continuing professional development in pediatric units could therefore consider integrating reflective practice and communication skills training with protected opportunities for emotional recovery, rather than relying on existing empathy alone.

### 4.4 Implications for healing workplace design and nursing management

This study offers three hypothesis-generating implications for healing workplace design and nursing management. First, pediatric units could consider including perceived personal accomplishment in routine well-being monitoring. Manager check-ins and staff well-being assessments should ask not only whether nurses feel exhausted, but also whether they feel recognized, competent, and able to grow professionally. Second, individual-resource interventions may be better evaluated as part of broader support packages rather than as stand-alone solutions. PsyCap training, mindfulness programs, and resilience workshops may have value, but future programs could pair them with attention to workload, scheduling, recognition, and career-development barriers. Burnout prevention programs could combine psychological support with workload review, transparent rostering, mentorship, and career-pathway planning. Third, continuing professional development should integrate reflective practice forums, peer debriefing, communication simulation, and compassion fatigue prevention, especially in NICU, PICU, and other high-acuity pediatric units. Training should be practical, confidential, psychologically safe, and grounded in real pediatric scenarios, consistent with the preferences expressed by nurses with extreme burnout profiles.

### 4.5 Limitations

Several limitations should be considered when interpreting these findings. First, the single-center design and the concentration of participants in NICU and PICU settings limit generalizability to other regions, hospital tiers, and lower-acuity pediatric services. This sample concentration may also have shaped the observed burnout profile and the non-significant PsyCap-burnout associations; replication in other pediatric settings with different organizational and staffing characteristics is needed to examine whether these patterns are context-specific. Because organizational variables such as workload, staffing levels, and leadership support were not directly measured, and the correlation analyses were unadjusted, interpretations about organizational conditions should be regarded as contextual inferences rather than quantitatively tested relationships. Second, the cross-sectional quantitative design precludes causal inference among burnout, PsyCap, and empathy, and longitudinal studies tracking nurses across career stages would strengthen understanding of how burnout dimensions and personal resources change over time. Third, because all measures were self-reported and collected from the same participants at a single time point, common-method variance and social desirability bias, particularly in empathy ratings, cannot be excluded. Anonymous administration, no collection of identifying information, and independence from supervisory relationships helped reduce this risk, but residual same-source bias cannot be excluded. Fourth, the single focus group included only nurses with extreme-burnout profiles, limiting transferability to the broader sample, nurse managers, and administrators. Group discussion of workplace-related burnout may also have constrained openness despite confidentiality and psychological-safety procedures, limiting the depth and diversity of qualitative findings. Finally, although the achieved sample of 218 nurses exceeded the 201-participant minimum derived from Slovin’s formula, future multi-site studies with formal *a priori* effect-size-based power planning would further strengthen the evidence base.

## 5 Conclusion

Pediatric nurses in this tertiary Chinese hospital exhibited a distinctive burnout profile characterized by a high prevalence of reduced personal accomplishment alongside comparatively low emotional exhaustion and depersonalization, low overall PsyCap, and high empathy. The absence of significant associations between PsyCap and burnout, set against the association of empathy with better perceived accomplishment and lower emotional exhaustion and depersonalization, suggests that the value of personal resources may vary across organizational contexts rather than being uniformly observed. Nevertheless, organizational conditions were not quantitatively tested, this interpretation should be considered exploratory. In high-intensity tertiary pediatric environments, the present findings suggest that organizational and professional support may warrant attention alongside individual psychological resources. Burnout management in such settings may therefore be considered as a multi-level approach that integrates individual psychological support with workload review, career-development support, team learning, and reflective practice, rather than as an individual-level training problem.

## Data Availability

The data that support the findings of this study are available from the corresponding author upon reasonable request.

## Footnotes

1
www.wjx.cn
