# Supplementary material for: When personal resources may not be enough: burnout, psychological capital, and empathy among pediatric nurses in a Chinese tertiary hospital — an explanatory sequential mixed methods study
Source: Front Public Health. 2026 Sep 9;14:1912710. doi: 10.3389/fpubh.2026.1912710 (PMC13597847; doi:10.3389/fpubh.2026.1912710)
Supplement: Supplementary file 1 [file Supplementary_file_1.docx]

**Supplementary Materials**

**Supplementary File 1**. The semi-structured interview guide

INTRODUCTION TO PARTICIPANTS

Thank you for taking part in this focus group discussion. Today we will spend approximately 90 to 120 minutes discussing your suggestions for burnout-related training and support. There are no right or wrong answers; we are interested in your honest views and practical suggestions. The discussion will be audio-recorded with your consent and transcribed verbatim. All identifying information will be removed during transcription, and the recordings will be securely stored and accessible only to the research team. You may decline to answer any question and may withdraw from the discussion at any time without consequence.

WARM-UP QUESTION

To begin, please briefly introduce yourself, including the unit you work in and how long you have been a pediatric nurse. There is no need to share any information you do not feel comfortable disclosing.

CORE QUESTIONS

The following six core questions guide the discussion. Optional follow-up prompts are provided to help the facilitator probe for depth where needed.

Q1. Have you ever attended any training or educational program on occupational burnout or work-related psychological support?

Probes:

• If yes, what topics were covered, and how was the training delivered?

• In what ways was the training helpful or unhelpful?

• If no, what kind of training would you have wanted to receive?

Q2. In your view, what content or topics should be included in a burnout-related training program designed for pediatric nurses?

Probes:

• Are there topics related to clinical work, emotional well-being, or career development that you consider particularly important?

• Are there issues specific to pediatric nursing that a generic program would not adequately address?

Q3. What teaching styles or educational strategies would you recommend for delivering this training?

Probes:

• What format works best for you, such as lectures, workshops, case discussions, online learning, simulation, or peer discussion?

• How long and how frequent should the sessions be?

• What conditions would make you feel safe and engaged in the training?

Q4. What suggestions do you have for the overall design and implementation of a burnout-related support and training program?

Probes:

• Who should be involved in delivering the training, such as psychologists, senior nurses, nurse managers, or peer mentors?

• What support would be needed to help nurses participate without adding to their workload?

• How could the program be evaluated to determine whether it is helping?

Q5. Of all the topics and ideas, we have discussed so far, which three do you consider the most important?

Probes:

• Why are these the most important to you personally?

• Do you think your colleagues would identify the same priorities?

Q6. What recommendations would you make to improve burnout-related support or mental health support for pediatric nurses in your hospital?

Probes:

• Are there current resources you find useful, and what could be improved?

• What forms of managerial or organizational support would make burnout-related training more feasible and acceptable?

CLOSING QUESTION

Is there anything important about burnout-related support, training needs, or program delivery that we have not yet discussed but that you would like to share?

───────────────────────────────────────

FACILITATOR NOTES

- Allow approximately 12 to 15 minutes for each core question.
- Where group consensus emerges quickly, invite participants who have not yet spoken to share their perspective.
- Where disagreement arises, encourage participants to elaborate on the reasons underlying their differing views, rather than resolving the disagreement.
- Maintain a non-judgmental tone throughout, particularly when participants describe negative experiences with management or colleagues.
- Conclude the session by thanking participants and reminding them of the confidentiality and data-handling procedures.

**Supplementary File 2.** Characteristics of focus group participants (n = 11)

| Participant  ID | Gender | Age (years) | Education | Department | Years of nursing experience | Professional role | Employment type | Monthly income (RMB) | Marital status |
| --- | --- | --- | --- | --- | --- | --- | --- | --- | --- |
| P1 | Female | 30 | Bachelor’s | Pediatric Neurology | 1 to <5 | Nurse | Contractual | 6000–9999 | Married |
| P2 | Female | 30 | Bachelor’s | NICU | 5 to <10 | Nurse | Contractual | 10000–19999 | Married |
| P3 | Female | 28 | Bachelor’s | Pediatric Neurology | 1 to <5 | Nurse | Contractual | 10000–19999 | Married |
| P4 | Female | 29 | Bachelor’s | NICU | 5 to <10 | Nurse-in-charge | Contractual | 6000–9999 | Married |
| P5 | Female | 34 | Master’s | PICU | 10 to <15 | Nurse-in-charge | Outsourced | 10000–19999 | Married |
| P6 | Female | 27 | Bachelor’s | Pediatric Neurology | 5 to <10 | Nurse | Contractual | 10000–19999 | Unmarried |
| P7 | Male | 25 | Bachelor’s | NICU | 1 to <5 | Nurse | Contractual | 6000–9999 | Married |
| P8 | Female | 29 | Bachelor’s | Pediatric Respiratory | 5 to <10 | Nurse | Contractual | 6000–9999 | Married |
| P9 | Female | 29 | Bachelor’s | NICU | 1 to <5 | Nurse-in-charge | Outsourced | 10000–19999 | Married |
| P10 | Male | 23 | Bachelor’s | Pediatric Infection | 1 to <5 | Nurse | Contractual | <6000 | Unmarried |
| P11 | Female | 24 | Bachelor’s | Pediatric Gastroenterology | 1 to <5 | Nurse | Contractual | <6000 | Unmarried |

Note. Participants were selected from nurses with extreme burnout profiles for the qualitative phase. Participants were coded P1–P11 to protect confidentiality.

Abbreviations: NICU, neonatal intensive care unit; PICU, pediatric intensive care unit; RMB, Chinese yuan/renminbi.

Monthly income ranges were reported in RMB. Approximate USD equivalents were calculated using 1 RMB = US$0.147 (May 7, 2026).
